# Acid-base disorders in critically ill neonates

**DOI:** 10.4103/0972-5229.68217

**Published:** 2010

**Authors:** S. Lekhwani, V. Shanker, G. Gathwala, N. D. Vaswani

**Affiliations:** **From:** Department of Biochemistry, Pt. B. D. Sharma PGIMS, Rohtak, Haryana, India; 1 Department of Pediatrics, Pt. B. D. Sharma PGIMS, Rohtak, Haryana, India

**Keywords:** Blood gas analysis, metabolic acidosis, plasma lactate

## Abstract

**Objective:**

To study acid–base imbalance in common pediatric diseases (such as sepsis, bronchopneumonia, diarrhea, birth-asphyxia etc.) in neonates.

**Design and Setting:**

An observational study was conducted in an emergency room of a tertiary teaching care hospital in Haryana, India.

**Patients and Methods:**

Fifty neonates (from first hour to one month) attending pediatric emergency services with various ailments. Blood gas analysis, electrolytes, plasma lactate, and plasma albumin were estimated in neonates.

**Results:**

Metabolic acidosis was the most common acid–base disorder. Hyperlactatemia was observed in more than half of such cases. Birth asphyxia was another common disorder with the highest mortality in neonates followed by bronchopneumonia and sepsis. Significant correlation between mortality and critical values of lactate was observed.

**Conclusion:**

Birth asphyxia with high-lactate levels in neonates constituted major alterations in acid–base disorders seen in an emergency room of a tertiary teaching care hospital. Plasma lactate concentration measurement provides an invaluable tool to assess type of metabolic acidosis in addition to predicting mortality in these neonates.

## Introduction

Understanding of acid–base dysfunction in various pathological conditions is an asset to a pediatrician in efficient treatment of critically ill children. The Henderson–Hasselbalch equation mathematically links the variables of pH, partial pressure of oxygen (*p*O_2_), partial pressure of carbon dioxide (*p*CO_2_), and bicarbonate concentration [HCO_3_^-^].[1] Blood gas analysis (BGA) provides pH, *p*O_2_, *p*CO_2_ from which [HCO_3_^-^] and base excess (BE) can be derived.[2] This approach has been considered “traditional.” Moreover, it is easily understandable and widely used at the bedside management.[3] Stewart’s so-called “modern” approach of electrical neutrality still requires explanation regarding applicability. Strong ion difference (SID), which is the difference between concentration of strong cations (Na^+^ and K^+^) and strong anions ([HCO_3_^-^] and lactate, is helpful in explaining the mixed-type acid–base disorders.[4] These two approaches had been the point of debate and comparison in several studies.[5–7]

Acid–base disorders reflect the seriousness of the underlying disease and are responsible for morbidity and mortality in sick children.[8] Marked structural and functional differences occur in children in comparison to adults, i.e., children have narrow distal airways, so atelectasis develops quickly resulting in rapid-onset of hypercarbia and hypoxia; chest wall is compliant and respiration is less efficient; the respiratory center is immature, hypoxia and hypercarbia lead to decreased respiratory drive. In addition, they have reactive vascular beds to maintain their blood pressure until late, so one cannot rely on hypotension to diagnose shock as in adults.[9] This study was carried out in neonates with various ailments attending the emergency room at a tertiary teaching care hospital of Haryana, India.

The objective was to study acid–base imbalance in common pediatric diseases (such as sepsis, bronchopneumonia, diarrhea, birth–asphyxia, etc.) in neonates (age, from first hour to 1 month).

## Patients and Methods

Fifty critically ill newborns attending emergency services were analyzed for acid–base status. Sample size was determined keeping 95% confidence level and 15% confidence interval considering the population of critically ill neonates requiring BGA as part of their management to be average 1200 attending emergency services round the year. Ethical approval was obtained from the Institutional Review Board since the study was carried out only on sick neonates, who require BGA report as part of the management by the pediatrician. No control group was taken as it would have been unethical to procure arterial samples of healthy neonates.

Heparinized arterial blood sample of patients with different pathological conditions was obtained at the time of admission before any intervention was made. Then, a 2 mL sample of heparinized arterial blood was obtained from each infant aseptically from the radial artery at the time of admission. As the volume of plasma required for the measurement of lactate (5 µL) and albumin (10 µL) was available as residue in the syringe after blood gas and electrolyte determination, the same was used for the measurement of lactate and albumin.[10]

Certain precautions were taken during the sample collection, such as usage of heparinized blood sample collection devices (syringes). As the BGA analyzer was equipped with Na^+^ sensor, NH_4_ ^+^ heparin was used instead of Na^+^ heparin. Radial artery puncture was preferred to avoid infection through the femoral artery because of close proximity to the groin area. Samples were taken in airtight, plastic, disposable syringes, which were flushed with heparin solution prior to sample withdrawal to avoid pH changes because of heparin. Plasma lactate was analyzed within 30 min of sample withdrawal to avoid possible changes in its values. BGA, electrolytes, plasma lactate, and albumin were analyzed using standard methods as follows:

*Plasma lactate* concentration was determined according to the following reaction,[11]

Lactate + O_2_ ^- Lactic oxidase^→ Pyruvate + H_2_O_2_

H_2_O_2_ + aminoantipyrine + TOOS -^Peroxidase^→ Purple product + 4H_2_O

where TOOS is *N*-methyl-*N*-(2-hydroxy-3-sulphopropyl)_*n*_-olivine.

The absorbance of dye complex was measured at 540 nm and is directly proportional to the lactate concentration in the sample.

Plasma was analyzed for lactate by kit (Randox Laboratories Ltd., Crumlin, Co. Antrim, United Kingdom) on a semi-auto analyzer (Transasia Bio-Medicals Ltd., Erba Diagnostics Mannheim, Germany). Samples were kept on ice after withdrawing the sample and transported and analyzed within 30 min of withdrawal. Although some authors recommend the use of sodium heparin for the sample collection,[12] ammonium heparin was used to avoid errors in the measurement of sodium for SID. As blood lactate levels are also affected by the use of lactated Ringer, samples were withdrawn before any intervention was made by the pediatrician.[13]

There were no exclusion criteria. Increased lactate levels due to conditions of excess production or diminished clearance; both were considered alike. Clinically diagnosed cases were not suggestive of any hepatic or renal impairment, so their organ function tests were not performed.

*Plasma albumin* was measured by bromocresol green (BCG) dye binding method.[14] BCG is yellow in undissociated form (pH > 4.7). At pH 4.1, i.e., below its *p*Ka of 4.7, only a small fraction is present in undissociated form. At pH 4.1, albumin acts as a cation and has binding-affinity toward the ionized BCG. Albumin binding at this pH with BCG forms a blue-colored complex which is proportional to albumin concentration. This color is measured at 620 nm.

Blood gas and electrolyte analyzer (Eschweiler modular) based on the principle of potentiometry analyzed pH, pO_2_, pCO_2_, Na^+^, K^+^, Cl^-^ by respective electrodes. BE and [HCO_3_ -] were calculated parameters from pH and pCO_2_, which were provided by the analyzer.

Anion gap was calculated from the following formula[15]:

AG = [Na^+^] + [K^+^] – [Cl^-^] – [HCO_3_ ^-^]

SID was calculated as follows[16]:

SID = [Na+]+ [K+] – [Cl^-^] –lactate

## Results

This study was carried out over a period of 3 months. The median (range) age of patients was 2 days (3 h to 1 month). The male-to-female ratio was 4.56:1 (41 males to 9 females). There was no correlation between either age or sex and severity of acid–base disturbances.

Table 1 gives a comparative observation of electrolytes and acid base parameters in major diagnosed pathological conditions among neonates (birth asphyxia and sepsis) while Table 2 shows the same among survivors and non-survivors.

**Table 1:** Acid base parameters (Mean ± S.D.) in birth asphyxia and sepsis

	Birth asphyxia (*n* = 21)	Sepsis (*n* = 11)
Na+ (mmol/L)	133.42 ± 8.01	146.7 ± 14.57
K+ (mmol/L)	5.16 ± 1.23	4.56 ± 1.85
CI- (mmol/L)	104.1 ± 15.57	119 ± 14.93
pH	7.338 ± 0.118	7.39 ± 0.048
pO_2_ (mm Hg)	97.95 ± 53.45	113.84 ± 68.44
pCO_2_ (mm Hg)	29.55 ± 11.94	25.92 ± 8.52
HCO_3_ (mmol/L)	15.24 ± 5.69	15.41 ± 4.87
H+ (mmol/L)	47.82 ± 13.88	40.31 ± 4.6
Lactate (mmol/L)	8.34 ± 9.14	3.49 ± 2.46
Albumin (gm/dL)	3.65 ± 0.67	3.52 ± 0.65
Anion gap (AG)	19.986 ± 14.15	16.85 ± 13.85
Strong ion difference (SID) (mmol/L)	26.27 ± 14.07	28.76 ± 10.14
Base excess (BE) (mmol/L)	-8.97 ± 6.54	-6.79 ± 4.085

**Table 2:** Acid base parameters (Mean ± S.D.) in survivors and non-survivors

	Survivors (*n* = 34)	Non-survivors (*n* = 16)
Na+ (mmol/L)	138.14 ± 13.4	138.69 ± 8.83
K+ (mmol/L)	4.84 ± 1.35	4.88 ± 1.45
CI- (mmol/L)	110.24 ± 17.33	111.25 ± 17.26
pH	7.39 ± 0.1007	7.21 ± 0.194
pO_2_ (mm Hg)	100.07 ± 55.78	102.78 ± 68.29
pCO_2_ (mm Hg)	26.63 ± 9.255	43.78 ± 31.57
HCO_3_ (mmol/L)	15.66 ± 5.28	15.525 ± 6.932
H+ (mmol/L)	41.74 ± 11.27	70.811 ± 48.11
Lactate (mmol/L)	4.66 ± 5.58	9.011 ± 9.88
Albumin (gm/dL)	3.68 ± 0.788	3.44 ± 0.74
Anion gap (AG)	18.28 ± 15.05	4.51 ± 27.04
Strong ion difference (SID) (mmol/L)	28.09 ± 9.54	18.54 ± 13.95
Base excess (BE) (mmol/L)	–7.02 ± 6.055	–13.24 ± 9.36

Significant correlation was found between outcome and critical values of pH (*P* < 0.01), *p*CO_2_ (*P* < 0.05) and lactate >2.5 mmol/L (*P* < 0.01). Of 50 neonates, 16 neonates died during their hospital stay. Raised blood lactate (>5 mmol/L) and lowest pH (<7.2) were associated with increase in patient mortality though worst BE (–10 mmol/L) had no association with survival.

Metabolic acidosis (BE ≤ –5mmol/L) was found in 34 patients. Hyperlactatemia was found in 97% and lactic acidosis was present in 35% of those suffering from metabolic acidosis. Birth asphyxia (21 patients) followed by sepsis (16 patients) was the major cause. Hyperchloremia (>120 mmol/L) was present in 26.5% of patients with metabolic acidosis. Increased albumin concentration was found in none. Decrease in pH was observed in 61.8% of such cases. Decreased SID (<38 mmol/L) was observed in 85.3% patients. SID had 85.3% sensitivity, 25% specificity, and 58% positive predictive value in detecting metabolic acidosis in neonates.

The sensitivity and specificity of pH, BE, and AG to detect hyperlactatemia were compared: BE was found to be 68.75% sensitive and 50% specific; pH 45.83% sensitive and 100% specific; and AG 54.2% sensitive as well as 50% specific to detect hyperlactatemia (>2.5 mmol/L).

## Discussion

**Figure 1:**
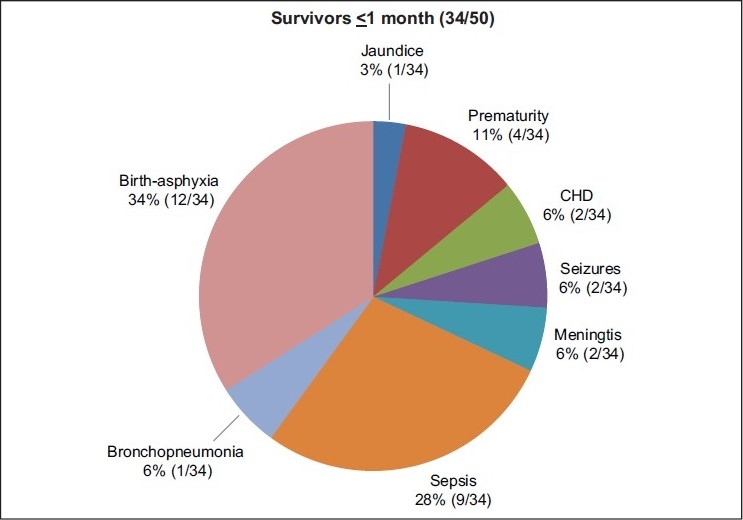
Incidence of various pathological conditions in survivors

**Figure 2:**
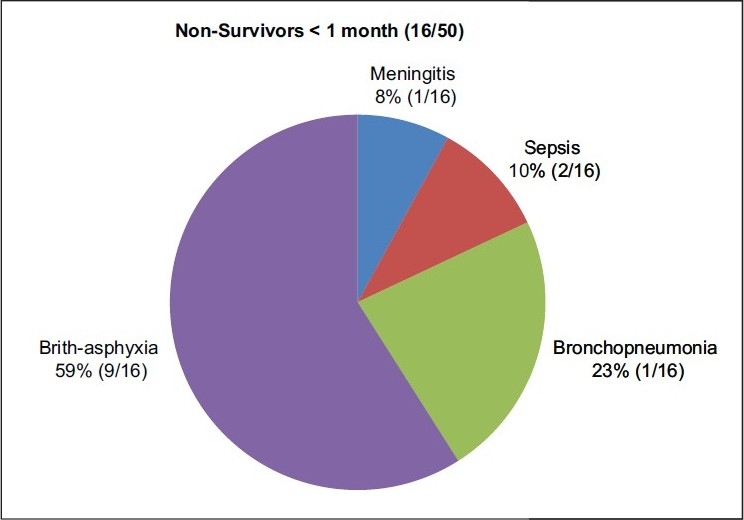
Incidence of various pathological conditions in nonsurvivors

The study was carried out on the basis of neonates suffering from a wide variety of ailments attending the pediatric emergency services [Figures 1,2]. The selection was unbiased. Preponderance of males in this age group, suffering from birth asphyxia is in accordance with the epidemiological pattern observed in this region. The data may, therefore, be generalized on a population of sick neonates seeking emergency care.

The acid–base status in major pathological disorders such as birth asphyxia, bronchopneumonia, sepsis, and diarrhea occurring in infants and neonates is discussed as follows.

In *birth asphyxia*, highest potassium levels [Table 1] reflected cellular changes because of diminished oxidative phosphorylation and ATP production. This energy failure impairs ion pump function, resulting in accumulation of intracellular Na+ and extracellular K+. Birth asphyxia presented with highest plasma lactate levels and lowest BE among all pathological conditions in the study [Table 1], and both had significant inverse correlation with each other (r = –0.547, *P* < 0.02). These findings are similar to da Silva et al.’s study in term neonates.[17] In this study, lower mean pH values were found in birth asphyxia. This is not surprising as previous studies show the same results. Palsdottir *et al*. evaluated the association between lower umbilical artery pH, more base deficit, and the development of hypoxic ischemic encephalopathy (HIE).[18] Chen *et al*. in their clinical study on improving the diagnostic criteria for neonatal birth asphyxia proposed umbilical artery blood pH < 7.00 as one of the integrated diagnostic criteria.[19]

Comparatively higher lactate levels were observed in sepsis. Hyperlactatemia is a cardinal finding in sepsis and septic shock. It is believed that the mechanism of hyperlactatemia for these two conditions is different. In sepsis, the increased lactate levels represent the increase glycolytic flux because of hyper metabolism, whereas in septic shock, the increase in glycolytic flux is because of hypoxia.[20] Here, metabolic acidosis was found to be compensated by respiratory alkalosis as inferred by positive correlation of BE with *p*CO_2_,[HCO_3_^-^], and pH. Sepsis has always been associated with hypoalbuminemia, and decreased albumin synthesis has been considered as a primary process in its causation.[21] Ruot *et al*. observed increased plasma albumin efflux contributing to hypoalbuminemia only during early phase of sepsis in rats.[22]

*Bronchopneumonia* presented with respiratory acidosis, while in some patients metabolic alkalosis with low albumin levels pointed out to the fact that respiratory acidosis may be compensated by metabolic alkalosis. Fencl *et al*. in their study on acid–base disorders in critically ill patients, found hypoalbuminemia as the only source of the severe metabolic alkalosis in one of the patients suffering from bronchopneumonia.[14]

### Despite diarrhea being a major health concern in infants, none of the neonates were seen with it in this study.

**Figure 3:**
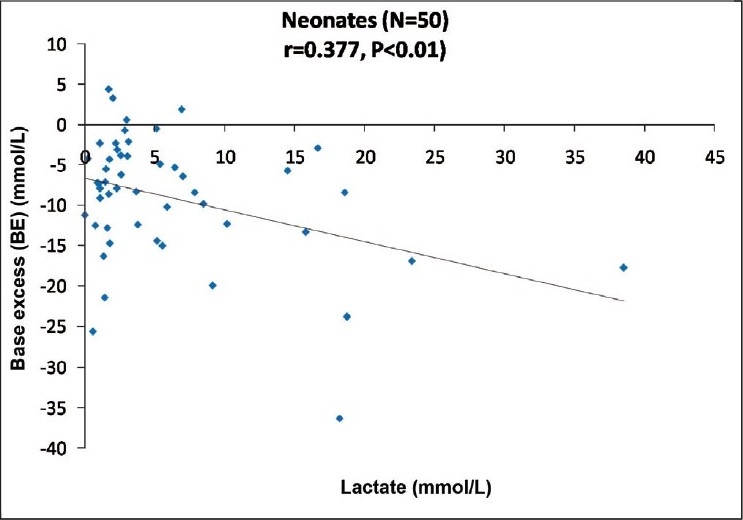
Correlation between standard base excess and lactate in neonates.

Hyperlactatemia was observed to be more severe in neonates. Neonates were unable to compensate metabolic acidosis by hyperventilating compared to infants as is clear from the finding that negative correlation was found between lactate and pH, also between lactate and BE in neonates [Figure 3].

Metabolic acidosis, according to the conventional concept (BE < –5 mmol/L),[24] suggests that 68% patients, whereas 82% patients suffer from the same if Stewart’s SID < 38 mmol/L[25] is considered as the criterion. This study has shown that metabolic acidosis is one of the most frequent acid–base disorders occurring in neonates, which is similar to Gauthier and Szerlip’s study on adults.[26]

The present study shows that plasma lactate and base deficit (negative BE) are higher in non-survivors, when compared with survivors [Table 2]. Changes in arterial pH and pCO_2_, but not BE are informative of the subsequent outcome (*P*<0.05 for pCO_2_, *P*>0.1 for BE). In this regard, measurement of blood lactate concentration (*P*<0.01), may give important prognostic information and an early warning signal.

**Figure 4:**
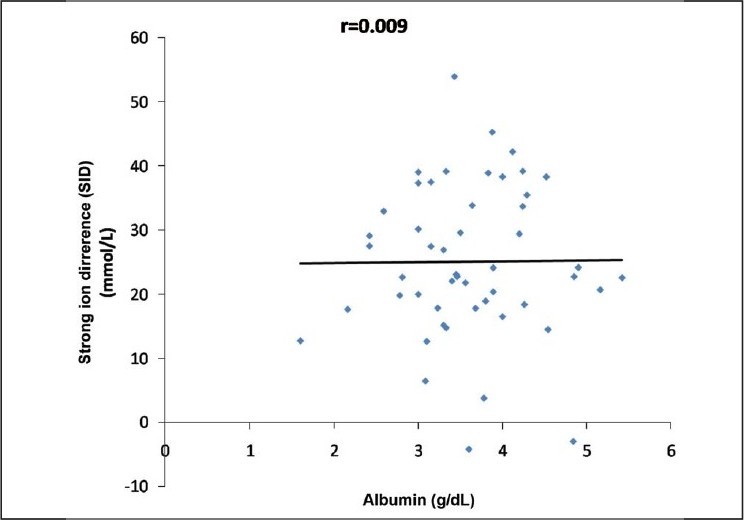
Correlation between albumin and strong ion difference.

Sirker considered metabolic acidosis to arise from conditions that cause either a reduction in the plasma SID or an increase in weak acids.[4] Albumin showed no correlation with pH, SID, AG, or BE in overall samples except significant positive relation with pH in sepsis [Figure 4]. Rossing *et al*. also could not find any correlation between SID and albumin.[26] This finding can be justified in this study as changes in protein levels usually occur in chronic diseases whereas in this case a wide variety of illnesses are dealt with in an emergency setup.

## Conclusions

This study on selected population of neonates affirms the fact that BGA, electrolytes, lactate levels provide valuable information for correction of acid–base imbalance associated with a pathological condition. Understanding the reasons behind such imbalance, both as a primary cause of critical illness and as a secondary complication, was the primary motive behind gathering together some aspects of acid–base disorders in selected pathological conditions. Albumin, a weak acid affects this balance in conditions like sepsis, where its levels decrease as it is lost into the extra-vascular compartment because of increased vascular permeability. Metabolic acidosis posing to be the most common disorder in this group of children, with lesser efficient compensatory mechanisms than those of adults, need more vigorous measures in an emergency situation. Traditional BE and AG still can be used in clinical settings to detect acid–base imbalance. Those disorders which could not be explained by these parameters require the concept of electrical neutrality, the SID.
